# Supplementary figures and images for: Developmental estrogen exposure in mice disrupts uterine epithelial cell differentiation and causes adenocarcinoma via Wnt/β-catenin and PI3K/AKT signaling
Source: PLoS Biol. 2023 Oct 19;21(10):e3002334. doi: 10.1371/journal.pbio.3002334 (PMC10586657; doi:10.1371/journal.pbio.3002334)

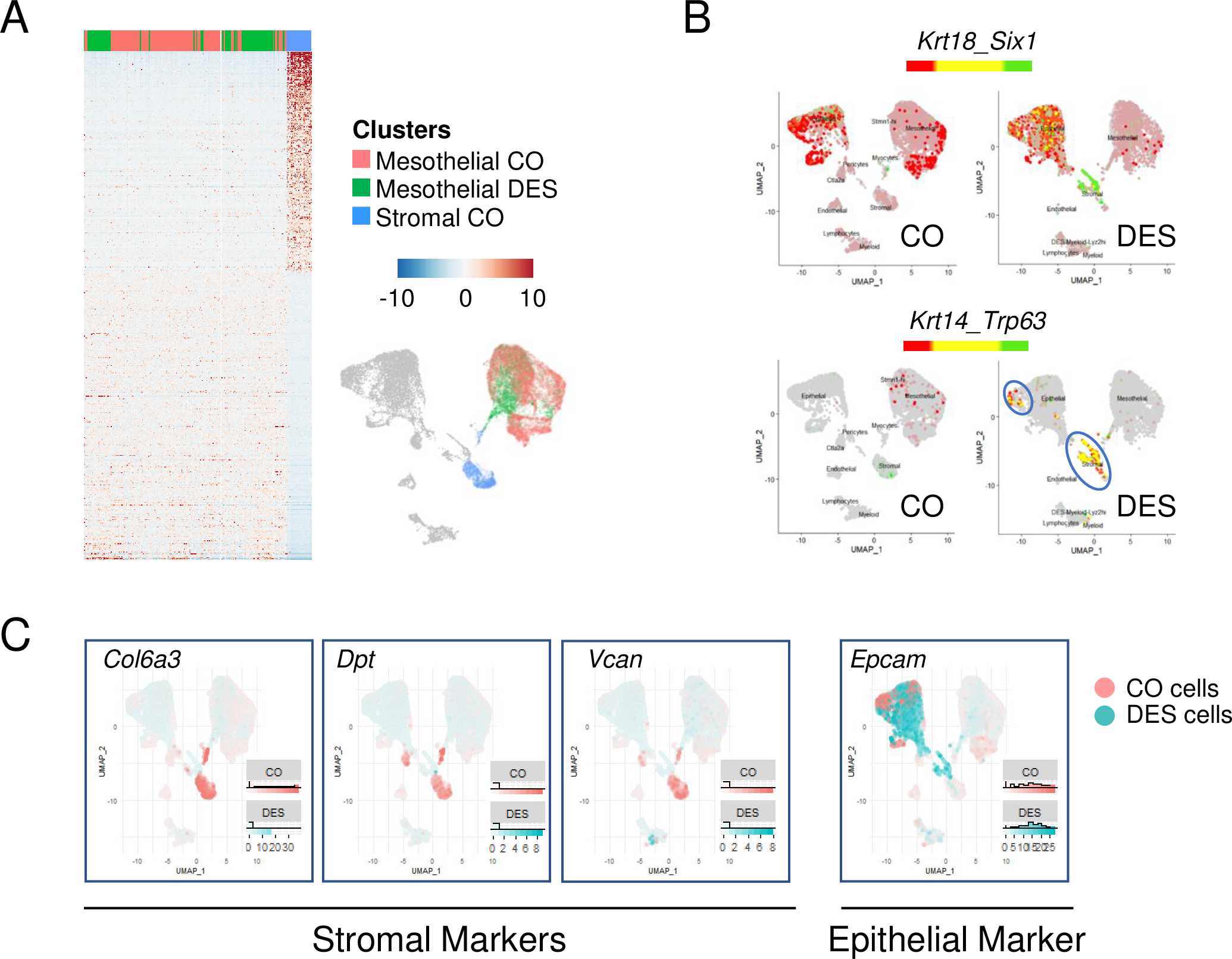

Supplement: S1 Fig — (A) Heat map of mesothelial and stromal cell markers in CO and DES epithelial cells. Expression is Pearson Residuals from the SCTransform method. UMAP is from Fig 1A with cell types indicated by color. (B) Dual feature plots of epithelial and basal cell markers (Krt18, Krt14, Six1, and Trp63). CO (left) and DES (right). Colors for each gene (red or green) are indicated above the UMAPs and yellow indicates overlapping expression. (C) Feature plots of stromal markers Col6a3, Dpt, and Vcan and epithelial marker Epcam using the integrated UMAP of all cells from CO (peach) and DES (teal). Expression is the same as in A. The data underlying this figure can be found in the Gene Expression Omnibus database under accession code GSE218156. (TIF) [file pbio.3002334.s001.tif]

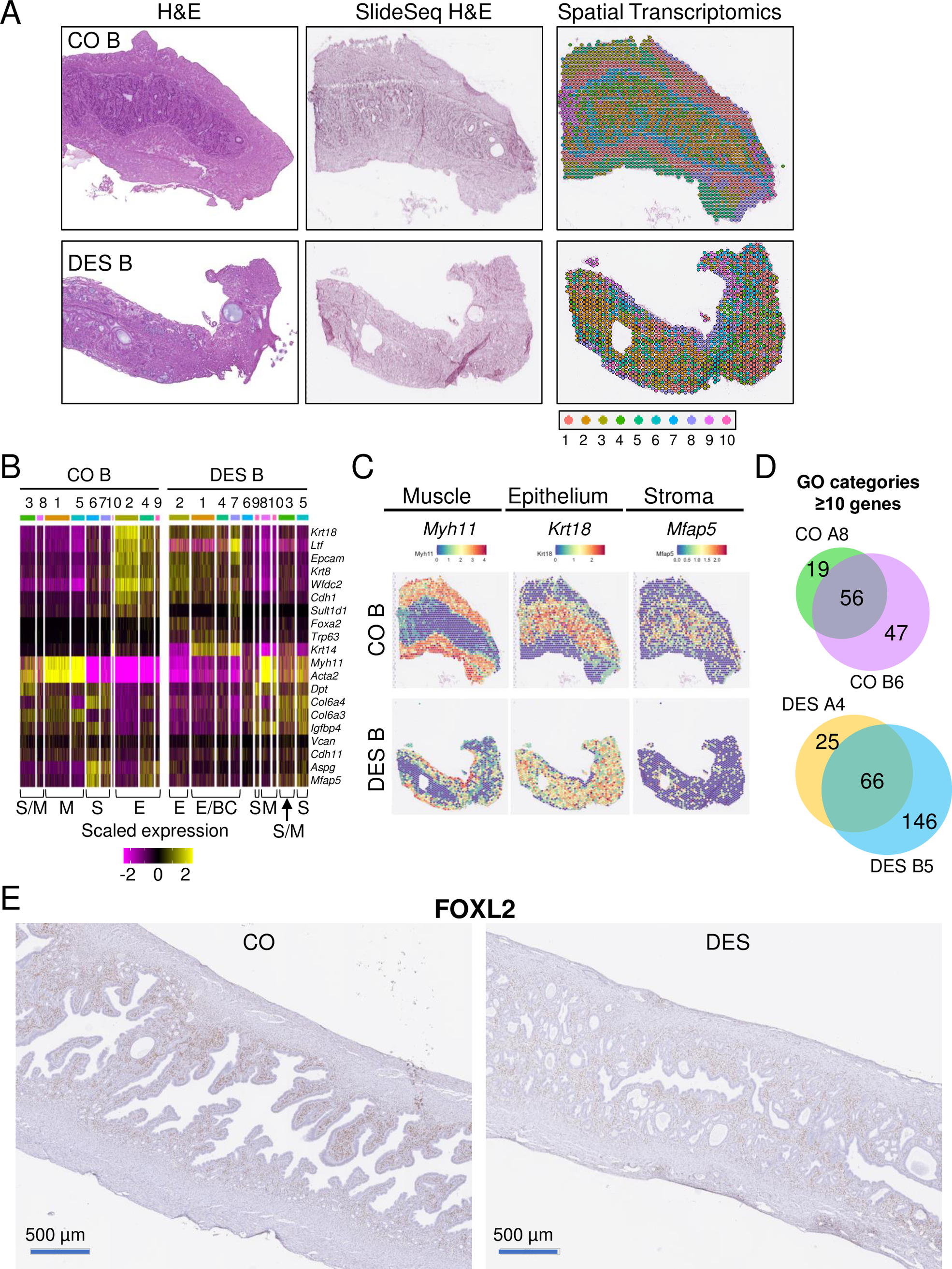

Supplement: S2 Fig — (A) Uterine tissue sections from CO B and DES B used for spatial transcriptomics (ST). HE stain of tissue section adjacent to ST section (left) and tissue sections used for ST (Slide-seq HE, middle). Cluster identification using Space Ranger-1.2.2, skmeans 10 (10x Genomics); colors represent distinct clusters. (B) Heat map of select uterine tissue cell type markers plotted for CO B (left) and DES B (right). Cell type is indicated below heat maps (M = muscle, S = stroma, E = epithelium, BC = basal cells). Expression values are Pearson Residuals from the SCTransform method. (C) ST section of select markers for CO and DES; expression = natural log transformed counts. (D) Venn diagrams of stromal cell GO categories with ≥10 genes (compared groups indicated). (E) Representative FOXL2 IHC in CO and DES; n = 4–6 mice per group. The data underlying this figure can be found in the Gene Expression Omnibus database under accession code GSE218156. (TIF) [file pbio.3002334.s002.tif]

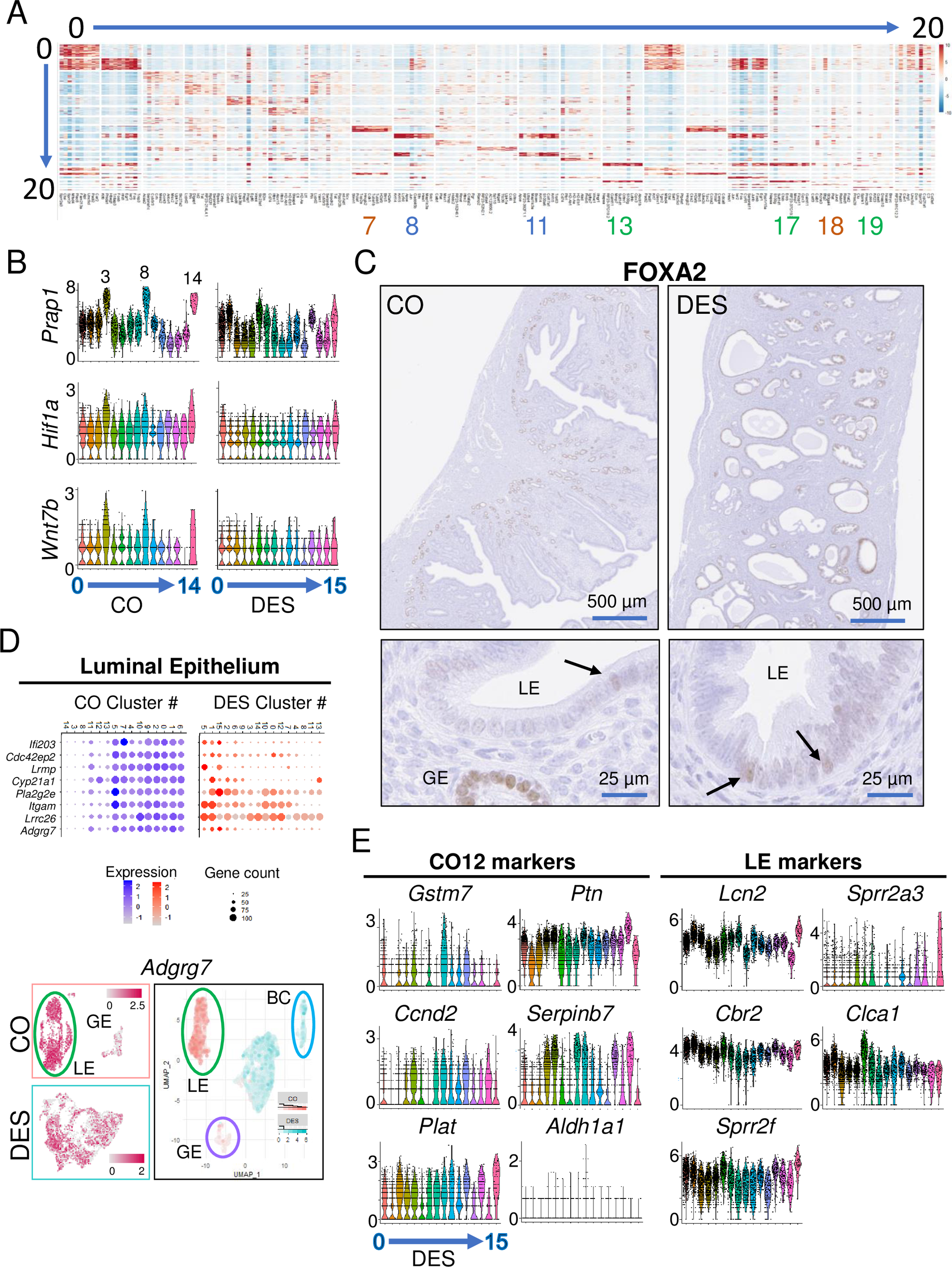

Supplement: S3 Fig — (A) Heat map of top DEGs of each cluster from integrated epithelial cell UMAP. Expression is Pearson Residuals from the SCTransform method. Clusters are indicated across the top and select clusters indicated: Basal cells (8, 11); GE (13, 17, and 19); overlapping CO and DES clusters (7 and 18). (B) Violin plots of developing GE markers (Prap1, Hif1a, and Wnt7b) for CO and DES. Cluster numbers are indicated below violin plots. Expression is natural log transformed counts and is indicated for each gene. (C) Representative FOXA2 IHC in CO and DES (n = 4–6 mice per group). LE and GE indicated. (D) Dot plots of 8 LE-specific genes in CO and DES epithelial clusters. Expression and gene count indicated. Feature plots of Adgrg7 expression in non-integrated CO and DES epithelial clusters as well as integrated CO and DES epithelial clusters. Expression levels are indicated. (E) Violin plots of top 5 CO12 and LE markers from Fig 3F in DES clusters. Cluster numbers are indicated below violin plots. The data underlying this figure can be found in the Gene Expression Omnibus database under accession code GSE218156. (TIF) [file pbio.3002334.s003.tif]

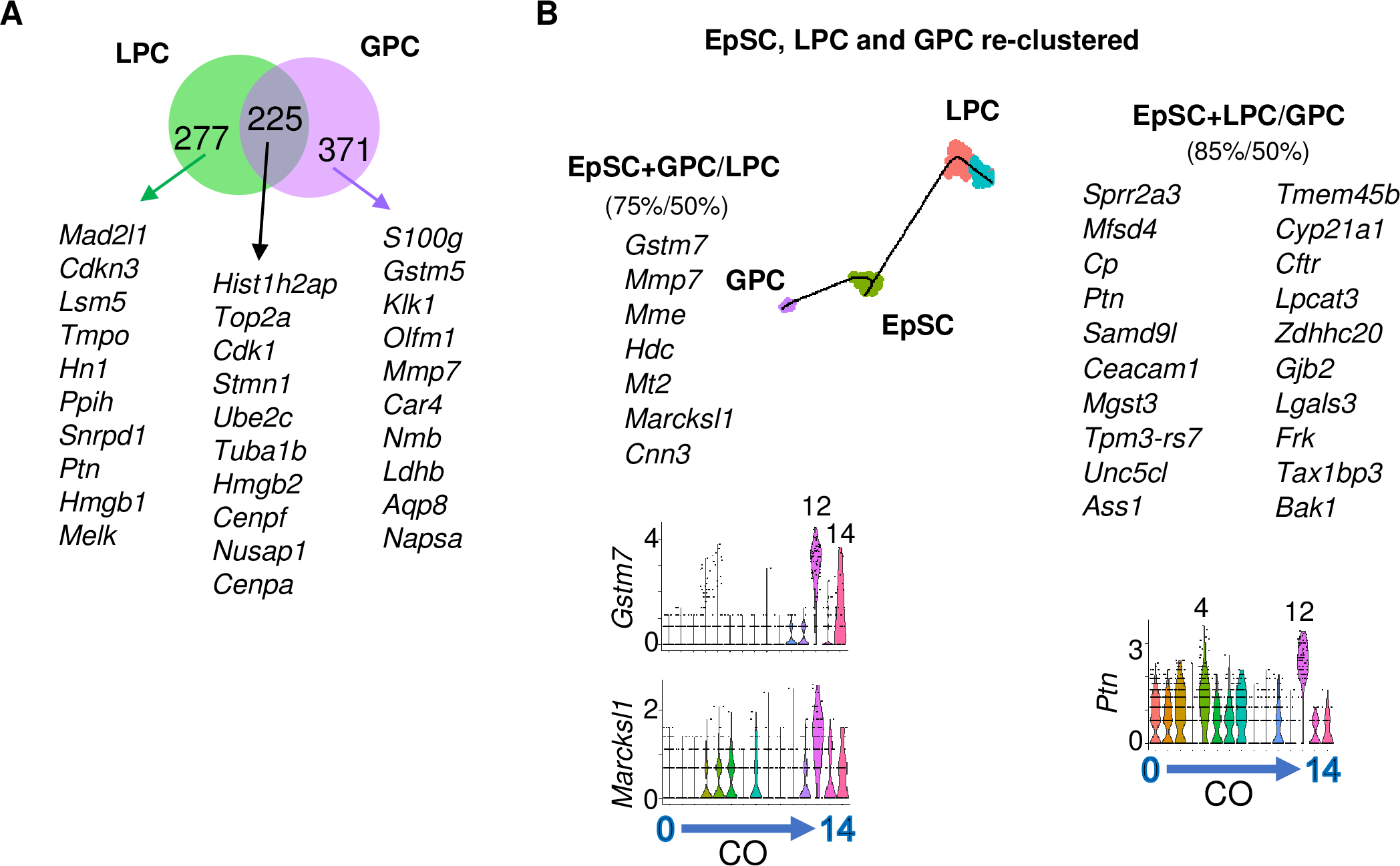

Supplement: S4 Fig — (A) Overlap of up-regulated LPC DEGs (CO cluster 4) with GPC DEGs (CO cluster 14). Ten highest fold-change DEGs in each category indicated. (B) Re-clustering of EpSC, LPC, and GPC with Slingshot analysis. Trajectory analysis using EpSC as the starting point showed 2 paths of differentiation (black lines). Top DEGs for each trajectory grouping (EpSC+GPC vs. LPC or EpSC+LPC vs. GPC); gene expression was filtered by the percent of cells expressing each DEG in each grouping compared to the remaining cluster; numbers in parentheses indicate the percent cells expressing in each group. Violin plots of CO epithelial clusters from Fig 3B of EpSC+GPC lineage genes, Gstm7 and Marcksl1, and EpSC+LPC lineage gene Ptn. Select clusters are indicated. Cluster numbers are indicated across the bottom. The data underlying this figure can be found in the Gene Expression Omnibus database under accession code GSE218156. (TIF) [file pbio.3002334.s004.tif]

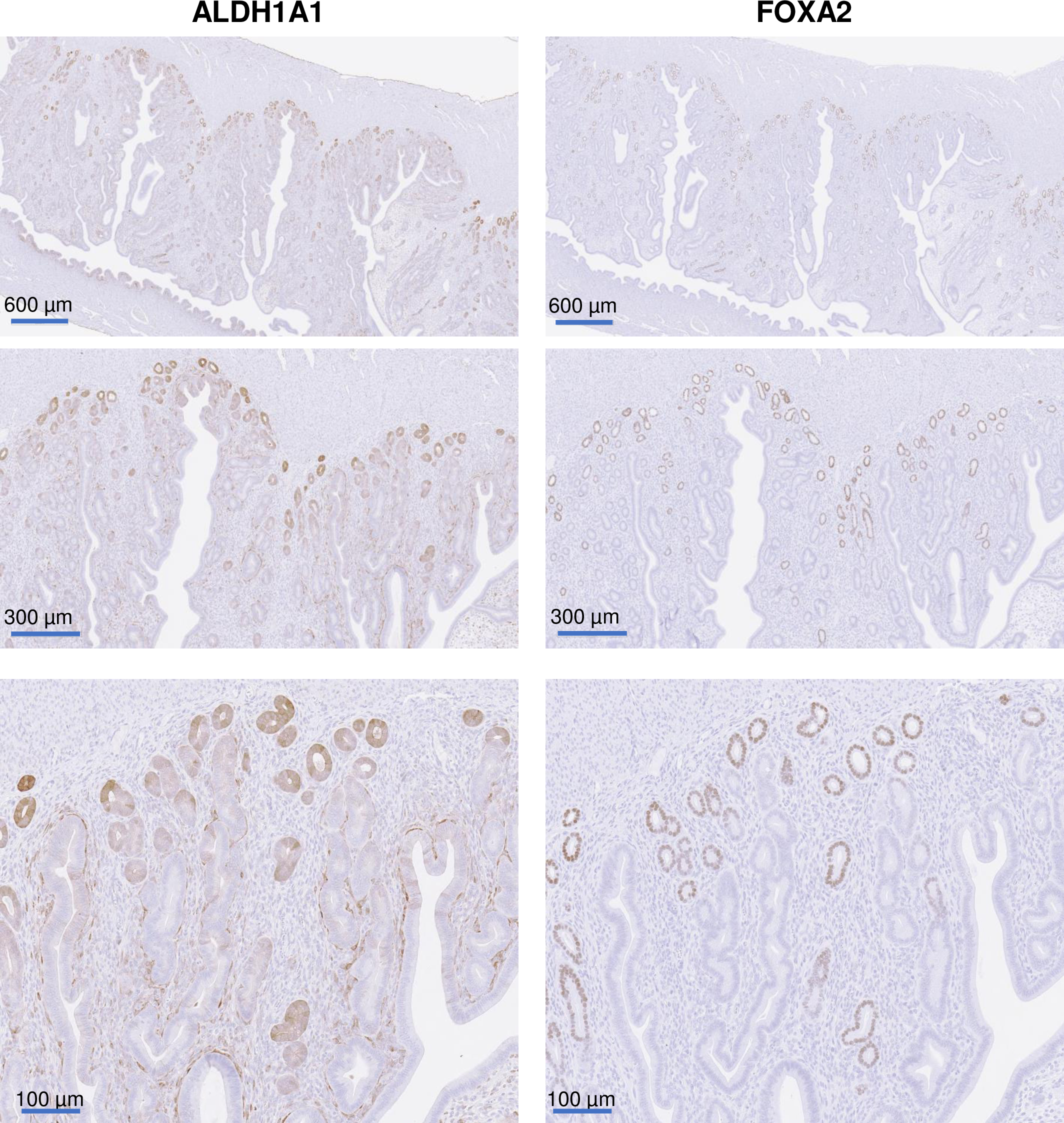

Supplement: S5 Fig — Representative ALDH1A1 and FOXA2 IHC in CO adjacent uterine sections (n = 4–6 mice per group). (TIF) [file pbio.3002334.s005.tif]

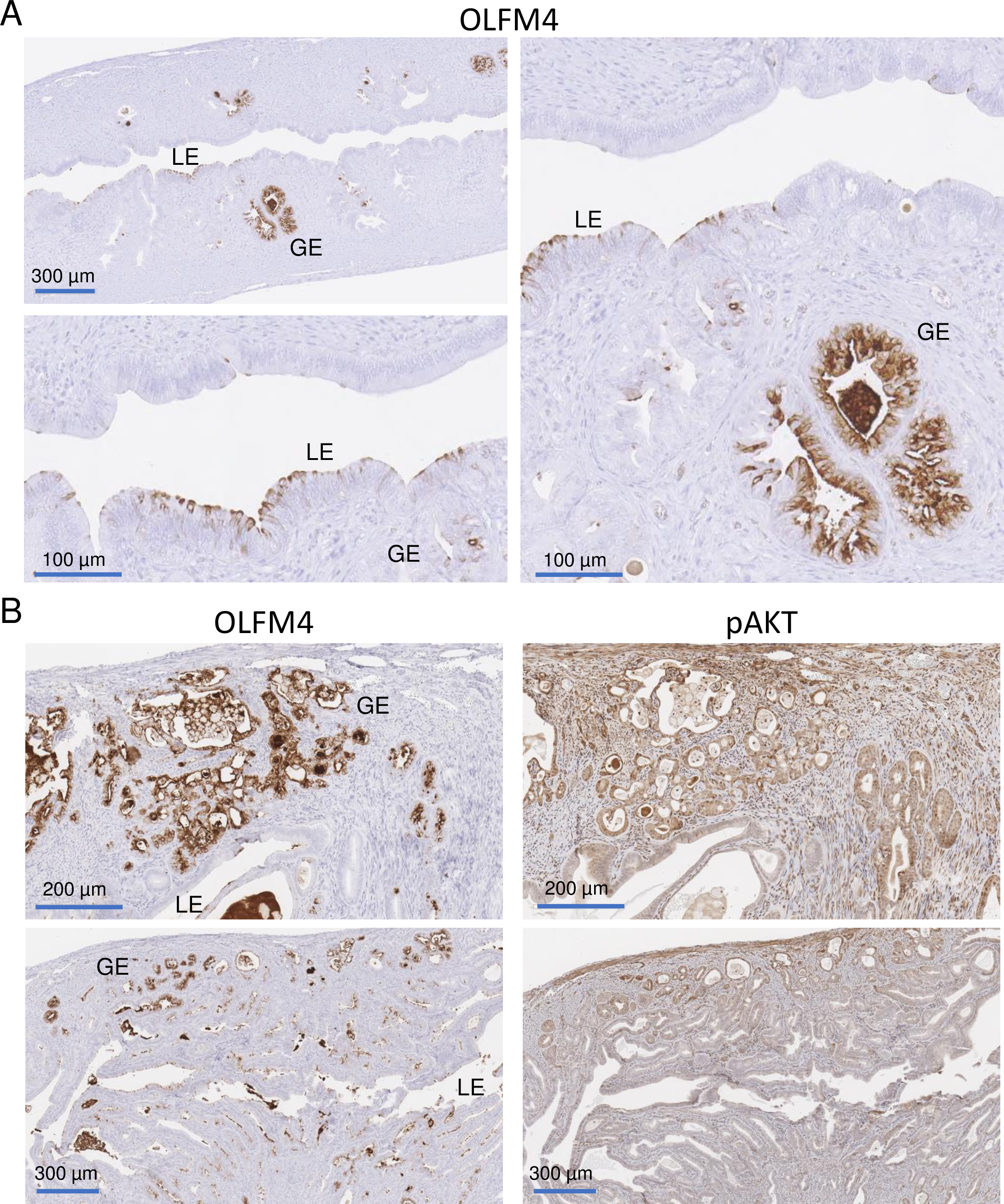

Supplement: S6 Fig — (A) Representative OLFM4 IHC in CD-1 12-month-old DES uteri (n = 4–6 mice per group). (B) Representative OLFM4 and pAKT IHC in adjacent sections from FVBN/J 9-month-old DES uteri (n = 4–6 mice per group). GE and LE are indicated. (TIF) [file pbio.3002334.s006.tif]

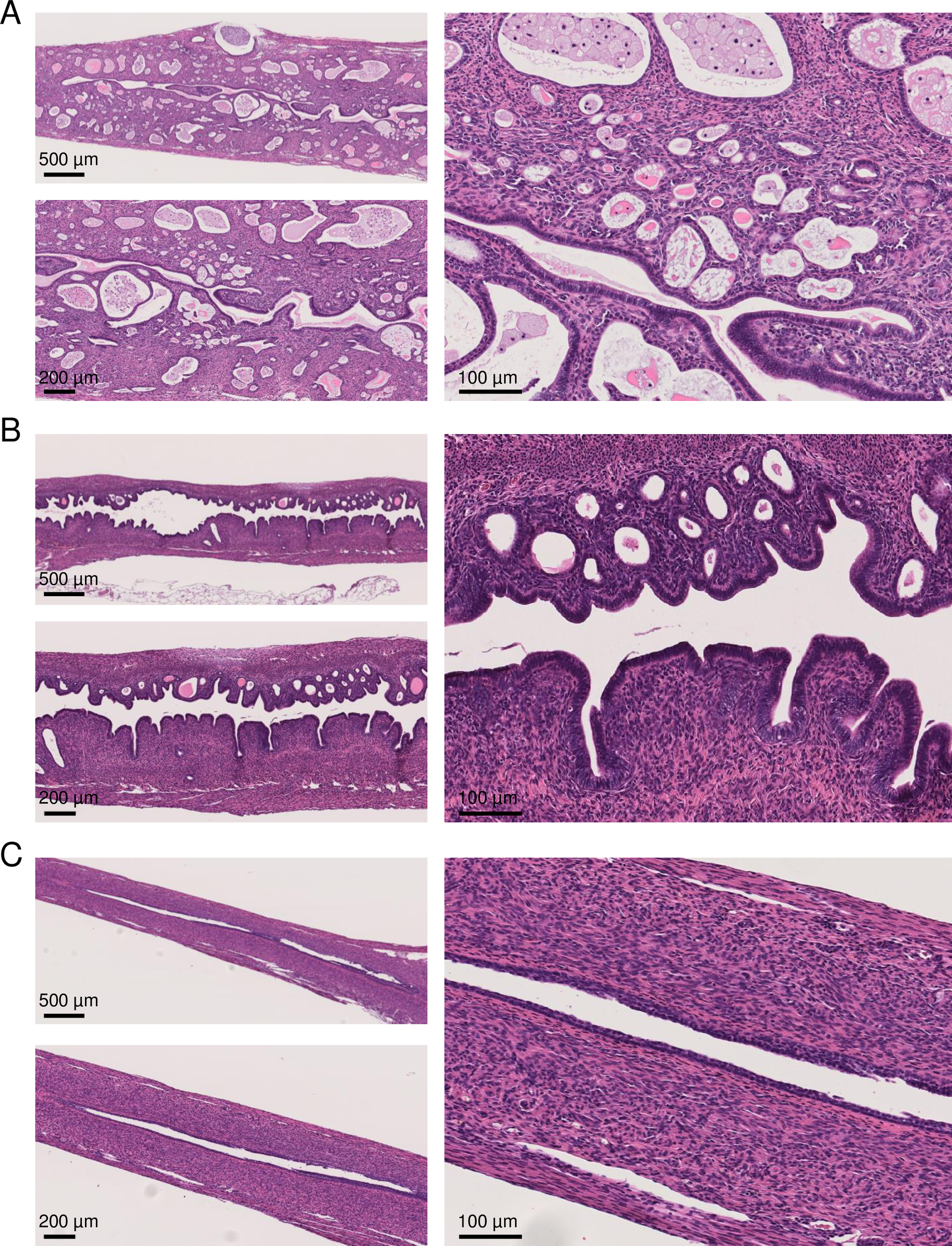

Supplement: S7 Fig — Representative HE staining of uterine tissue sections from 12-month-old mice exposed neonatally to DES (n = 4–6 mice per group). (A) Esr1-flox/flox (wild type). (B) Esr1-flox/flox; Amhr2-cre+ (stromal ERα cKO). (C) Esr1-flox/flox; Wnt7a-cre+ (epithelial ERα cKO). (TIF) [file pbio.3002334.s007.tif]

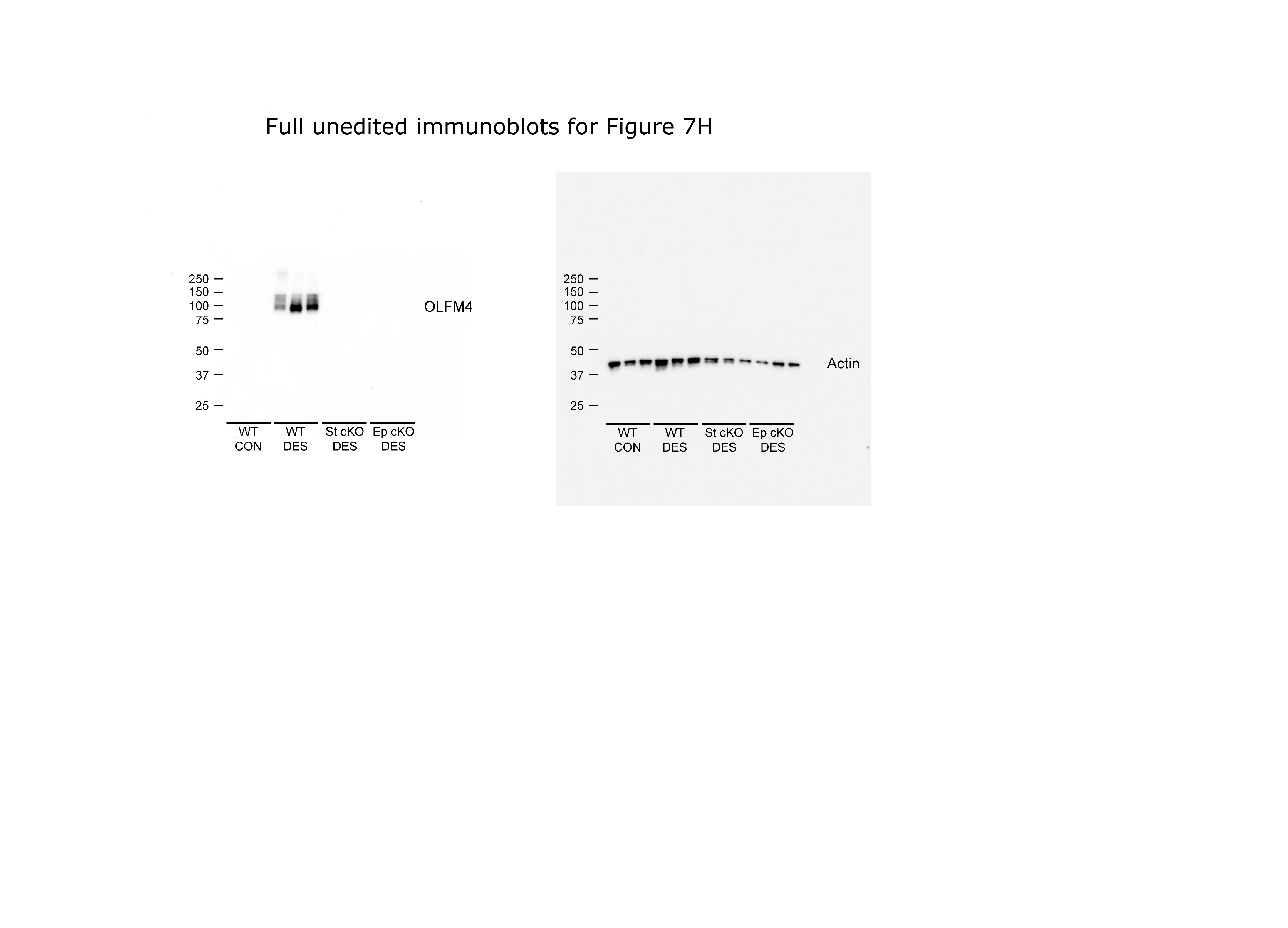

Supplement: S1 Raw Images — Left: Immunoblot of OLFM4 in uteri from 12-month-old control or DES-treated mice. Each lane has 5 μg uterine protein extract from 1 mouse (n = 3 mice per group). Right: Same blot as in left panel reprobed for actin. Molecular weight markers to left of each blot (kD). WT, wild type; St cKO, stromal ERα conditional knockout; Ep cKO, epithelial ERα conditional knockout. (TIF) [file pbio.3002334.s008.tif]
