# Supplementary material for: Developmental estrogen exposure in mice disrupts uterine epithelial cell differentiation and causes adenocarcinoma via Wnt/β-catenin and PI3K/AKT signaling
Source: PLoS Biol. 2023 Oct 19;21(10):e3002334. doi: 10.1371/journal.pbio.3002334 (PMC10586657; doi:10.1371/journal.pbio.3002334)
Supplement: S12 Table — (DOCX) [file pbio.3002334.s020.docx]

**Supplemental Table S12. KEY RESOURCES TABLE**

| REAGENT or RESOURCE | SOURCE | IDENTIFIER |
| --- | --- | --- |
| Antibodies | | |
| Rabbit monoclonal anti-Aldh1a1 | Abcam | Cat# ab52492; RRID: AB_867566 |
| Rabbit polyclonal anti-En2 | Sigma-Aldrich | Cat# AV31375; RRID: AB_1848149 |
| Goat polyclonal anti-Foxl2 | NOVUS Biologicals | Cat# NB100-1277; RRID: AB_2106187 |
| Rabbit monoclonal anti-Olfm4 (D6Y5A) XP | Cell Signaling Technology | Cat# 39141; RRID: AB_2650511 |
| Mouse Monoclonal anti-Topo II alpha (G-6) | Santa Cruz Biotechnology | Cat# sc-166934; RRID: AB_10611755 |
| Rabbit polyclonal anti-Phospho-Akt (Ser473) | Cell Signaling Technology | Cat#9271; RRID: AB_329825 |
| Rabbit monoclonal anti-FoxA2/HNF3 (D56D6) XP | Cell Signaling Technology | Cat# 8186; RRID: AB_10891055 |
| Monoclonal rabbit IgG | Abcam | Cat#ab125938; RRID: AB_2924650 |
| Rabbit on rodent HRP polymer | Biocare Medical | Cat#RMR625 |
| Biotinylated goat anti-rabbit IgG | Vector Laboratories | Cat#BA-1000; RRID:  AB_2313606 |
| Biotinylated horse anti-goat IgG | Vector Laboratories | Cat# BA-9500; RRID:  AB_2336123 |
| Normal rabbit IgG | Millipore Sigma | Cat#N101-100UG; RRID: AB_2924651 |
| ImmPRESS HRP Horse anti-rabbit IgG Polymer | Vector Laboratories | Cat#MP-7401; RRID:  AB_2336529 |
| Mouse monoclonal anti-beta actin peroxidase | Sigma-Aldrich | Cat#A3854; RRID:AB_262011 |
| Peroxidase donkey anti-rabbit | Jackson ImmunoResearch Labs | Cat# 711-035-152, RRID: AB_10015282 |
|  |  |  |
| Bacterial and virus strains | | |
|  |  |  |
| Biological samples |  |  |
|  |  |  |
| Chemicals, peptides, and recombinant proteins | | |
| Diethylstilbestrol (DES) | Sigma-Aldrich | Cat#D4628; CAS#56-53-1 |
| Phosphate buffered saline, calcium and magnesium free (PBS-CMF) | NIEHS media preparation | N/A |
| Trypsin-EDTA (0.25%), phenol red | Gibco-Thermo Fisher | Cat#25200072 |
| DMEM/F-12, HEPES | Thermo Fisher Scientific | Cat #11330-032 |
| CellTrics 30 µm, sterile | Sysmex, Fisher Scientific | Cat#NC9682496 |
| CellTrics 100 µm, sterile | Sysmex, Fisher Scientific | Cat#NC1037263 |
| Trypan Blue | Thermo Fisher | Cat#15250061 |
| Tissue-Plus O.C.T compound | Fisher Scientific | Cat#23-730-571 |
| Methanol | Millipore Sigma | Cat#34860 |
| Mayer’s hematoxylin | Millipore Sigma | Cat# MHS16-500ML |
| Eosin | Millipore Sigma | Cat# HT110216-500ML |
| 10% neutral buffered formalin | NIEHS Media preparation | N/A |
| Xylenes | Fisher Scientific | Cat#X5-4  CAS#1330-20-7 |
| Hydrogen peroxide | Fisher Scientific | Cat#AC426001000  CAS#7722-84-1 |
| Heat inactivated fetal bovine serum | Thermo Fisher | Cat#10-438-026 |
| Bovine Serum Albumin | Thermo Fisher | Cat#11020-021 |
| 2-Propanol | Fisher Scientific | Cat# 02-002-099  CAS#67-63-0 |
| Acetic acid | Millipore Sigma | Cat#A6283 |
| Tris Base | Thermo Fisher | Cat#BP152-500 |
| Shandon Bluing reagent | Thermo Fisher | Cat#6769001 |
| RiboLock RNase inhibitor | Thermo Fisher | Cat#E00382 |
| EDTA solution | Biocare Medical | Cat#ORI6006T60 |
| Rodent Block M | Biocare Medical | Cat#RBM961 |
| Isopentane | Millipore Sigma | Cat #270342 |
| Diaminobenzidine (DAB) | Agilent | Cat#K346811-2 |
| Normal goat serum | Jackson ImmunoReserach | Cat#005-000-001 |
| Normal horse serum | Jackson ImmunoResearch | Cat# 008-000-001 |
| HALT | Thermo Scientific | Cat# 1861281 |
| TPER | Thermo Scientific | Cat# 78510 |
| Precision Plus Protein Dual Color molecular weight markers | Bio-Rad | Cat#1610374 |
| 10% TGX Stain free Gels | Bio-Rad | Cat# 4568036 |
| Tris/Glycine/SDS buffer | Bio-Rad | Cat# 1610732 |
| Transblot Turbo Transfer kit | Bio-Rad | Cat#17001919 |
| 10X TBS | Bio-Rad | Cat#1706435 |
| Tween-20 | Sigma Aldrich | Cat#9005-64-5 |
| Blotto, non-fat dry milk | Santa Cruz | Cat#sc-2325 |
| SuperSignal West Femto kit | Thermo Scientific | Cat#34095 |
| 2-mercaptoethanol | Sigma | Cat#M7154 |
|  |  |  |
| Critical commercial assays | | |
| Chromium Single Cell 3’ Reagent Kit v3 | 10X Genomics | Cat#1000075 |
| Visium Spatial Gene Expression Slide kit | 10X Genomics | Cat#1000184 |
| Avidin-Biotin blocking kit | Vector Laboratories | Cat#SP-2001 |
|  |  |  |
|  |  |  |
| Deposited data | | |
| Raw RNA-seq and spatial transcriptomics data | This paper | GEO: GSE218156 |
|  |  |  |
| Experimental models: Cell lines | | |
|  |  |  |
| Experimental models: Organisms/strains | | |
| Adult CD-1 mice | NIEHS/NIH in house breeding colony | Crl:CD1(ICR) |
| Adult FVBN/J mice | Jackson Labs | FVBN/J; Jax # 001800 |
| Adult Esr1 flox (FVB) mice | Kenneth Korach, NIEHS; FVBN/J background (>10 generation backcross); FVB;B6-Esr1<tm4.1>Ksk | N/A |
| Adult Amhr2-cre (FVB) mice | Francesco Demayo, NIEHS; FVBN/J background (>10 generation backcross); FVB;129S7-Amhr2<tm3(cre)Bhr>/ Mmnc | N/A |
| Adult Wnt7a-cre (FVB) mice | Kenneth Korach, NIEHS; FVBN/J background (>10 generation backcross); FVB;B6-Tg(Wnt7a-EGFP/cre)?Bhr | N/A |
|  |  |  |
| Oligonucleotides | | |
|  |  |  |
| Recombinant DNA | | |
|  |  |  |
| Software and algorithms | | |
| 10x Genomics Cell Ranger 3.0.1 | doi.org/doi:10.1038/ncomms14049 [1] | https://support.10xgenomics.com/single-cell-gene-expression/software/pipelines/latest/installation |
| Seurat v3.1.0 | [doi:10.1016/j.cell.2019.05.031](https://doi.org/10.1016/j.cell.2019.05.031) [2]  [doi:10.1038/nbt.4096](https://doi.org/10.1038/nbt.4096) [3]  [doi:10.1038/nbt.3192](https://doi.org/10.1038/nbt.3192) [4] | https://satijalab.org/seurat/articles/install.html |
| 10x Genomics Space Ranger 1.2.2 | 10x Genomics | https://support.10xgenomics.com/spatial-gene-expression/software/pipelines/latest/installation |
| Slingshot v2.2.1 | doi.org/10.1186/s12864-018-4772-0 [5] | https://bioconductor.org/packages/release/bioc/html/slingshot.html |
| scran v1.22.2 | doi.org/10.12688/f1000research.9501.2 [6] | bioconductor.org/packages/release/bioc/html/scran.html |
| DropletUtils v1.14.2 | [doi.org/10.1186/s13059-019-1662-y](https://doi.org/10.1186/s13059-019-1662-y) [7]  doi.org/10.1038/s41467-018-05083-x [8] | https://bioconductor.org/packages/release/bioc/html/DropletUtils.html |
|  |  |  |
| Other | | |
|  |  |  |

**References**

1. Zheng GXY, Terry JM, Belgrader P, Ryvkin P, Bent ZW, Wilson R, et al. Massively parallel digital transcriptional profiling of single cells. Nat. Commun. 2017;8.

2. Stuart T, Butler A, Hoffman P, Hafemeister C, Papalexi E, Mauck WM, et al. Comprehensive Integration of Single-Cell Data. Cell 2019;177:1888–1902.e21.

3. Butler A, Hoffman P, Smibert P, Papalexi E, Satija R. Integrating single-cell transcriptomic data across different conditions, technologies, and species. Nat. Biotechnol. 2018;36:411–20.

4. Satija R, Farrell JA, Gennert D, Schier AF, Regev A. Spatial reconstruction of single-cell gene expression data. Nat. Biotechnol. 2015;33:495–502.

5. Street K, Risso D, Fletcher RB, Das D, Ngai J, Yosef N, et al. Slingshot: Cell lineage and pseudotime inference for single-cell transcriptomics. BMC Genomics 2018;19:1–16.

6. Lun ATL, Mccarthy DJ, Marioni JC. A step-by-step workflow for low-level analysis of single-cell RNA-seq data with Bioconductor [ version 2 ; referees : 3 approved , 2 approved with reservations ]. F1000Research 2016;5:1–69.

7. Lun ATL, Riesenfeld S, Andrews T, Dao TP, Gomes T, Marioni JC. EmptyDrops: Distinguishing cells from empty droplets in droplet-based single-cell RNA sequencing data. Genome Biol. 2019;20.

8. Griffiths JA, Richard AC, Bach K, Lun ATL, Marioni JC. Detection and removal of barcode swapping in single-cell RNA-seq data. Nat. Commun. 2018;9.
