# Supplementary material for: Developmental estrogen exposure in mice disrupts uterine epithelial cell differentiation and causes adenocarcinoma via Wnt/β-catenin and PI3K/AKT signaling
Source: PLoS Biol. 2023 Oct 19;21(10):e3002334. doi: 10.1371/journal.pbio.3002334 (PMC10586657; doi:10.1371/journal.pbio.3002334)
Supplement: Attachment — Submitted filename: Response to reviewers 060823.docx [file pbio.3002334.s022.docx]

We are grateful to all three reviewers for their helpful comments and corrections and believe that the manuscript has been strengthened through this process. We have addressed all concerns in a revised version of the manuscript. In the responses below, the line numbers refer to the “track changes” version.

Of note, Brian Papas has now been included as a co-first author rather than as a middle author due to his efforts carrying out the additional bioinformatics analyses required for this revision.

**Reviewer #1:** In the current manuscript, the authors applied scRNAseq and spatial transcriptomics to map the differentiation of endometrial epithelia, and reveal the developmental exposure of DES as a big risk of adenocarcinoma progress in adult caused by dysregulation of uterine epithelial differentiation. The findings are really novel and advancing our understanding of stem cell and cancer biology in the uterus. Here are some minor comments for authors to revise the manuscript:

1. A published paper (Reference 17) used lineage tracing to identify the differentiation of epithelial stem cells at a cellular level, in which the stem cell population is located in the intersection area between luminal and glandular epithelia. Here, the authors applied scRNAseq and spatial transcriptomics to further define this differentiation process by mapping the dynamic changes of genes at each stage during the whole epithelial lineage differentiation as well as their location. This map of epithelial differentiation serves as the guidance of cyclical endometrial regeneration, a foundation of uterine health and disease including cancer. This finding is a significant advance to the reproductive biology field. However, the current title of this manuscript does not reflect this key point. A better title is needed to cover both differentiation of  epithelial stem cells and cancer formation caused by developmental exposure of DES.

Response: The title has been changed to include this aspect of the findings, which we agree better represents the findings in the manuscript.

2. Figure 3G labeling was missing.

Response: Sorry for any confusion, there is no panel 3G. The reference in the manuscript should have been 3F and has been corrected (Line 333).

3. Figure 7 legend was mixed in the discussion part.

Response: This is a summary figure that is not referenced until the Discussion. The journal requests that we put the figure legend after the paragraph that first mentions the figure.

4. A specific marker for luminal epithelia in the mouse uterus is lacking currently, it will be a big contribution to the field if authors can further analyze the sequencing data to define a specific marker of LE in the future studies. This comment is just for the future study.

Response: We agree that having specific markers of LE cells would be useful, so we went ahead and performed an additional analysis to address this question. We generated a restricted set of DEGs that were expressed in >75% of the cells in the large control LE cluster and were expressed in <15% of the remaining cells (excluding CO12). This analysis resulted in 8 LE-specific genes. We have added this information in the Results and included a new figure panel in the Supplemental figures (Figure S3D and Lines 313-325).

**Reviewer #2:** Padilla-Banks et al. use single-cell transcriptomics to characterize an endometrial cancer mouse model based upon diethylstilbesterol (DES) exposure.  Overall, the impact of the paper is high given both the sharp increase in endometrial cancer in women and lack of in vivo models that recapitulate endometrial carcinogenesis.  Of particular importance is the links identified between DES exposure and known oncogenic and tumor suppressor pathways in endometrial cancer, including PI3K pathway activation with developmental estrogen exposure.  Therefore, I support consideration at PLOS Biology and have the following specific comments.

1)      The TP53 mutations and/or loss are common in the uterine serous carcinoma, not endometrioid subtypes.  In addition, TP53 mutations often don't co-exist PIK3R1 mutations in human endometrial cancer.  Most human endometrial cancers without identifiable molecular alterations are TP53 wild-type.   Although this does not change the importance of the findings or translational impact, especially in how it pertains to PI3K pathway activation, these details should be clarified in the text and further discussed, since it's likely the DES-model represents a rare or uncommon form of endometrial cancer.  Thus, it may limit the use of the DES-model in the exploration of common forms of endometrial cancer.

Response: Because TP53 mutations are far more common in uterine serous carcinomas, even though they are present in 25% of the endometrioid subtype (Getz G et al., Nature 2013;497:67–73), to avoid confusion we have removed TP53 from our discussion of the implications of our findings for human endometrial cancer. The main parallel between the DES model and human endometrioid cancers is activation of PI3K/AKT signaling, which is now better highlighted with removal of TP53 from this Discussion paragraph. We have also emphasized that most human endometrial cancers are associated with mutations, unlike the case in the DES model (Lines 740,744-745).

2)      The mouse sample sizes used for the single cell studies need to be properly justified.  Based on the methods, both control and DES-exposed mouse uteri were pooled from individual mice, then processed for library preps and sequencing.   It seems odd to pool samples from individual mice, rather than individually examine each mouse in the treatment groups.  It's also unclear if the orthogonal assays were performed on the same set of treated mice or if additional treatment groups were analyzed.  Overall, the authors should attempt to show that the molecular mechanisms by which DES-exposure promotes endometrial carcinogenesis are recapitulated in the models described.

Response: We agree that ideally, multiple individual samples are independently sequenced to obtain the most robust data. However, to obtain sufficient living cells for the single cell RNA-seq we needed more than one uterus per sample, so pooling uteri from several mice was necessary to get the numbers we needed. Single cell sequencing at the depth we used was cost prohibitive for multiple individual samples, so we used one pooled sample for each group. The single cell RNA-seq, spatial transcriptomics and IHC experiments were all performed on independent groups of mice. This approach allowed us to validate key findings in different mice by distinct methods. We have clarified the Methods section to address these questions (Lines 786-787).

**Reviewer #3:** This study composed an extensive and deep single-cell (sc) transcriptomic atlas of the adult mouse uterus in control and DES-exposed conditions, and extracts interesting findings from it. The dataset will be very useful for the endometrium research community.
The study presents a bioinformatic tour de force with application of multiple cutting-edge computational tools. It touches on the (further) characterization of epithelial stem and progenitor cells of both luminal and glandular epithelium. Interestingly, cell types and clusters are completely different in the DES-exposed uteri and it took original and elaborate approaches to be capable of comparing control with DES conditions.

Nevertheless, there are still a number remarks and questions, and addressing (some of) them will further strengthen the study.


Introduction
The reason of exposure to DES could be a bit more situated, in view of its past clinical use and associated severe side effects.

Response: We now provide in the Introduction a more detailed description of outcomes of human fetal DES exposure and point out that the mouse DES model was developed to model these outcomes (Lines 40-43, 53-54).

Results
Line 90: " more than 1.2 billion reads per sample": is this very large number correct?

Response: Yes – these numbers are correct. These two samples were sequenced very deeply resulting in >1.2 billion reads per sample (CO: 1,449,861,325 and DES: 1,202,821,237) .

Line 91: The number of CO cells in the scRNA-seq dataset is double the number of DES cells, while uteri from 4 DES mice were pooled compared to only 2 CO mouse uteri. How do the authors explain this? Due to the loss of stromal cells from DES uteri? Could the cell isolation protocol from DES uteri be further optimized not to lose these cells? Then, stromal cells could be analyzed in the scRNA-seq data without the need for spatial transcriptomics.

Response: When the single cell RNA-seq was performed, the only methodology available was to use living cells that were processed immediately. It took more than one uterus per sample to get enough cells, so we pooled uteri from several mice to get the numbers we needed. In addition, we had to collect control mice that were in estrus on the day of the experiment, to match the persistent estrus of the DES-exposed mice, and we had only two mice in estrus from the 12-month-old controls on the day of collection for this experiment. (Note that we had a limited number of mice to choose from because they must be aged to 12 months and at this age, even control mice do not all cycle regularly). For DES, the uteri are smaller (hypoplastic) compared to controls and because the mice do not cycle, we could pool as many as we needed. In addition, it was more difficult to isolate living cells from DES-exposed mice than was true for control tissue. We believe that this is explained by the fact that neonatal DES exposure results in excess extracellular matrix deposition in the uterine endometrial stroma and muscle layers, and the tissue is stiffer. Four DES mice gave us the correct number of living cells for this experiment. We tried many isolation protocols to capture high enough quality living cells for this experiment, but unfortunately the one included in the manuscript was the only method that worked.

We are in the process of performing additional single cell RNA-seq experiments on younger mice to obtain a timeline of differentiation changes. For these studies, we are using the newer method of sequencing isolated nuclei from frozen tissues, which we believe will alleviate the problem of the stiff DES tissue. We anticipate getting nice representation of stromal cells this way.

Line 119-122: DES mesothelial cell gene expression instead of DES epithelial cell gene expression is shown in Fig. S1A; is this a mistake in the text?

Response: Yes, thank you. The text was corrected (Line 135).

Line 125: Basal cells are mentioned; hence this cell type needs much more explanation here. In addition, it is only mentioned in the Discussion that these cells are normally not present in the uterus. Should be described earlier in the Results sections.

Response: We have now added to the Introduction a paragraph that provides context for the basal cell phenotype in the uterus following neonatal DES exposure (Lines 77-86).

Line 146: Spatial transcriptomic analysis was eventually done for only 1 section per condition. How reproducible are the findings? Sections from additional (independent) mouse uteri should be subjected to spatial transcriptomics and analyses included.

Response: We had initially considered repeating the spatial transcriptomics on independent sections, but because the 55-µm resolution of the method was quite limiting for our evaluation of specific cell populations in the context of the endometrial stroma and epithelium, we did not feel it was worth the expense and effort. To address the reviewer’s concern that only one section was used per condition, we revisited the second CO and DES sections from the original slide. Although these sections were not as high quality as the two sections we used in the original version of the manuscript, we were able to use them to test whether our major findings were replicated in independent mice. Analysis of the replicate spatial transcriptomics data provided similar information to the initial sections evaluated and we now have added it to the Results (Lines 192-212), Supplemental Table (S3H-N), and Supplemental Figure S2A-D.

Line 186-188: Can the authors provide potential explanations on how DES exposure results in the altered phenotype (including the disrupted expression pattern of Foxl2), regarding e.g. altered signaling pathways by DES, or increased inflammation in stromal cells?

Response: This is a very good question that we are unable to definitively answer because our data are from only a single time point after the cancer is already formed. We are currently performing scRNA-seq on control and DES-exposed mice at several earlier time points to address this question. We speculate that neonatal DES exposure alters the stem/progenitor cell population and that these changes result in altered differentiation/cancer in response to later endogenous estrogen exposure, but we need more evidence to support this idea. We feel it would be too speculative to include this information in the current manuscript.

Line 200-201: Fig. 3A seems contradictory to Fig. 1A. In Fig. 1A, DES and CO epithelial cells largely overlap in the UMAP plots. Hence, they would be expected to have similar transcriptomics/gene expression. However, the "overlapping" CO and DES epithelial cells are not present in Fig. 3A? Please clarify.

Response: Integration for Figure 1A was performed to remove CO and DES differences to make cell types more apparent. We used sample integration following Seurat’s CCA integration vignette for Figure 1A (and only for that Figure). The UMAP in Figure 3 is limited to epithelial cells selected based on their expression of *Krt18*. Because we were no longer including the wide variety of cell types found in the uterus, large differences in the epithelial cell populations became apparent in the new UMAP. We used Seurat v3.1.0 following the standard SCTransform-based pipeline with MAST for Figure 3A to allow for differences in CO and DES epithelial cells. We have added this information to the Methods section (Lines 823-826).

Line 226: should be Fig. S3A instead of Fig S2A?

Response: Yes, thank you. The text was corrected.

Line 227: Foxa2 is not included here. Why?

Response: *Foxa2* is not a gland specific gene as there is some expression in the luminal epithelium. It also was not in the top 10 DEGs for each category for cluster 13, 17 or 19.

Line 238: "The lack of overlap in UMAP locations of CO and DES epithelial cells". Their original UMAP (Fig. 1A) clearly shows large overlap of epithelial cells of both groups. Did the authors consider possible 'batch effects' with variations in sample groups due to technical influences rather than biological differences? Please provide more details on the integration methods used in both experiments.

Response: See response regarding Line 200-201 above. We used sample integration following Seurat’s CCA integration vignette for Figure 1A (and only for that Figure) and Seurat v3.1.0 following the standard SCTransform-based pipeline with MAST for Fig 3A to capture differences. We have added this information to the Methods section (Lines 823-826). Because the library preparation and sequencing were performed at the same time, batch effects cannot explain these differences.

Line 287: Fig. 3F instead of Fig. 3G

Response: Yes, thank you. The text was corrected.

Lines 358 and 461: CON should be CO. The latter should be used consistently, also in all figures.

Response: We have changed all instances of CON to CO.

Line 297: For the Slingshot analysis, how was the starting point chosen? Does this occur in an unbiased manner by the program, or does the researcher indicate this starting point? In the latter case, the trajectory may be biased. Please clarify.

Response: For a Slingshot analysis, the researcher indicates the cluster that is the likely starting point. In this case, we had already identified our progenitor population with published markers and used this cluster as the starting point for the glandular epithelium trajectory. The resulting single trajectory clearly passed through all other clusters. Directionality of the trajectory from the Slingshot analysis can be a the starting or ending point of the line. Based on the presence of progenitor cell markers at one end and mature gland markers at the other, directionality was clear.

For the new luminal epithelial cell cluster trajectory, we similarly chose the progenitor cell cluster as the starting point. In this case, there were 7 distinct trajectories, but all had to either arise from or end in the progenitor cells, and it would not make sense for the trajectories all to end at the same point. For this reason, we believe that we correctly identified the starting point for this Slingshot analysis as well.

We have added information in the Results section for clarity (Lines 344-345).

Authors elaborate on the GE clusters and their trajectory, but neglect the LE cells in this respect. What are the DEGs/specific markers of LE, and how is the time trajectory within this population? In addition, authors tentatively indicate GE and LE progenitor cells and epithelial stem cells (e.g. 350-353). What is the relationship between these? Pseudotime trajectories should also be determined here. In the end, functional support for stem and progenitor phenotypes is needed by, for instance, lineage tracing using the newly defined markers. Please add to the Discussion.

Response: We have now performed a more complete analysis of the LE cell population, including identification of DEGs, LE-specific markers, and a trajectory analysis and included this new information in the Results section (new Fig 5 and panel S3D, and new Supplemental Tables S8A-B, Lines 388-397). We also explored the relationship between the EpSC, LPC and GPC. DEGs were compared between LPC and GPC and we performed a trajectory analysis of the 3 clusters. The trajectory analysis provided DEGs specific to each trajectory. These findings have been included in the Results section (new Fig S4A-B, Supplemental Table S10A-B, and Lines 411-421, 447-457). Finally, the need for validation of our newly identified stem and progenitor cell markers has been added to the Discussion (Lines 669-671).

Lines 397-401 etc: it is strange that the authors aim at testing the identification of the stem and progenitor cells using their identified marker ALDH1A1 and then show its expression in mature GE. This does not fit at all. Should be better and further explained.

Response: We used *Aldh1a1*, *Lgr5*, and *Axin2* as potential markers of the stem cell population based on reports in the literature that we referenced. Most of these studies were performed in neonatal mice, so information on potential adult epithelial stem cells was limited. When combining the expression of these three markers with the absence of cell division markers, as the stem cells should not be dividing rapidly, we determined the identity of the EpSC population. The expression of *Aldh1a1* in the distal tips of the expanding GE has been published (Seishima et al., 2019) and we confirmed that here. Our findings suggest that the genes specifically expressed in CO12 such as *Gstm7* (Table S6C) could be markers of EpSC (Fig 3F and S4B). We have added clarification in the Results section (Lines 411-413; 447-457).

Lines 450-451: Six1 was used as basal cell marker (see Fig. 1D), but here it is used as cancer marker in the non-basal cell DES clusters. This is not comprehensible, and should be clarified. Moreover, from the feature plot (Fig. 6A), Six1 is not "most highly expressed in DES cluster 5" as the authors state. High expression is also present in many other DES clusters. Please clarify and adjust if needed.

Response: We have added information to the Introduction to explain *Six1* expression in both basal cells and uterine cancer cells and its use as a biomarker for uterine cancer in both mice and humans. We recognize that there is high expression of *Six1* in other DES clusters, but we used the highest expressing cluster as the potential cancer cluster. The two DES clusters that have significantly increased *Six1* were DES clusters 5 and 14. *Six1* was 1.55-fold increased in DES cluster 5 and 1.44-fold in DES cluster 14. The significantly increased expression of *Rad51b* as a DNA damage marker in DES cluster 5 but not DES cluster 14 confirmed our identification of DES cluster 5 as the cancer cell population. We have added this information to the text for clarification (Lines 532-533).

Line 489: Wnt signaling may be driving (contributing to) the DES cancer phenotype. Are there mouse models (e.g. beta-cat mutant) that support this?

Response: Yes, there are models that capture different aspects of the DES phenotype. One model we described in the Discussion is where β-catenin is constitutively activated in intestinal epithelial cells. It is characterized by de-differentiation of the epithelial cells and a gain of stem cell like properties. This model also activates the inflammatory response. In addition, a model of constitutive uterine activation of β-catenin results in uterine hyperplasia. This model combined with PTEN loss accelerates uterine cancer formation. A model of conditional uterine deletion of β-catenin results in squamous cell hyperplasia (basal cell phenotype). We have added this information to the Discussion (Lines 713-716).

Line 492: Fig. 5F à There is no panel F in Fig. 5. Should be Fig. 5E?

Response: Yes, you are correct. This has been changed to new Fig 6E (previously 5E) in the text.

Line 503: OLFM4 staining is visible in the cavity of the glands (see Fig. 6D and Fig. 6F). Is this background staining or is this secreted protein? Please clarify.

Response: The appearance of the brown staining in the glands in now Figures 7D and 7F does resemble secreted protein, but we do not believe this is the case. If you zoom in, you can see a cobbled appearance that indicates these were likely cells. The cell borders in these lumens probably lost their definition during processing. Cells in lumens are a common finding in DES exposed mice at this age. They are more easily identified in the OLFM4 IHC in Figure S6B and in the adjacent pAKT IHC in Figure 7G, and they appear to express OLFM4. We originally thought they were an immune cell type, but by IHC they were negative for multiple immune cell markers including CD45. These cells remain unidentified. We now have added text in the Results to include these cells (Lines 589-591).

Line 537-539: it is suggested that the stromal inflammation and oxidative stress influences the epithelium. It could also be possible that the abnormal differentiation of epithelial cells induces stromal inflammation and oxidative stress. Please discuss. Moreover, a graphical abstract of the main findings would be clarifying for the reader.

Response: This point is a good one, and it was the reason we did the experiment using DES-exposed stromal ERα cKO and epithelial ERα cKO mice. If abnormal differentiation of epithelial cells was the cause of stromal inflammation and oxidative stress, then we would expect there to be cancer in the stromal ERα cKO. However, in both cKO models, no cancer developed, suggesting that DES-induced changes in both epithelium and stroma are necessary for the cancer phenotype. This point is made in the Results (Lines 632-640). It will be interesting to determine the timeline of neonatal DES exposure-induced changes in stromal and epithelial cell gene expression across development, which we are pursuing currently.

We have included a graphical abstract to capture the main findings of this manuscript, as well as a working model of control and DES-induced epithelial cell differentiation (Figure 8).

Line 546-547: it is strange that here (Fig. 6H) OLFM4 immunoblotting is used and not staining of sections. It reads as a major gap that no uterine sections of these genetic mouse models are shown. Does histology clearly show the absence of cancer? These analyses should be added.

Response: The uterine tissues from the 12-month-old conditional stromal and epithelial deletions of ERα were very thin and upon sectioning, we only had a few that had enough luminal epithelium to evaluate, so a thorough histological assessment of cancer incidence could not be done on these mice. However, we have now added to the results the descriptions of some of the available tissue sections from each of the models that clearly show cancer in controls and the lack of any cancer formation in both cKOs (Lines 632-640) and Supplemental Fig S7A-C).

Line 588-590: If the basal cells do not contribute to the cancer phenotype, what do the authors suggest to be their contribution to the DES phenotype?

Response: We have added information in the Introduction about the basal cell phenotype and that SIX1 controls this part of the aberrant differentiation. Lack of SIX1 eliminates the formation of basal cells in the uterus but this does not preclude uterine cancer formation, suggesting that basal cells do not play a major role in the cancer phenotype (Lines 77-86).

Figures:
Line 110: Fig. 1C comment: Top 25 (said in legend) versus Top 50 (shown in figure)

Response: We have added text in the Legend for clarification. The Top 25 for epithelial cells + top 25 for stromal cells resulted in a total of 50 Top DEGs.

In Fig. 1A, green is used for epithelial cells, in Fig. 1C, green is used for stroma. Could be made more uniform.

Response: We selected the colors for Fig. 1C to effectively highlight the cell clusters being shown. We chose not to use green for the epithelium because in Fig. 1A, green is used for all epithelial cells and in Fig. 1C, the epithelial cells are split into CO and DES. We could not identify shades of green that would effectively show the locations of CO cells distinctly from DES cells. It seemed to us that it would be more confusing to match some but not all colors.

Fig. 1C: What does the title "DES" refer to? The UMAP and heatmap plots show both DES and CO.

Response: The incorrect residual DES label has been removed from this part of the figure.

Fig. 1C left: Epithelial markers are much lower in DES epithelial cluster compared to CO epithelial cluster. However, in Fig. 1D KRT18 expression appears equally high between CO and DES. Please provide a scalebar in Fig. 1D to show the relative expression between the two groups.

Response: Dual feature plots were used in Fig. 1D to identify which clusters contained basal cells and which contained non-basal epithelial cells, so a comparison of expression levels is not needed here. Instead, it is necessary to have relatively saturated signal for both red and green colors where the respective gene is highly expressed so that co-expression in the same cell will appear yellow.

It is interesting that overall, the expression level of epithelial marker genes seems lower in DES compared to CO. We have added this observation in the text (Lines 139-140). However, *Krt18* seems to be an exception. A comparison of *Krt18* in CO epithelial cells and DES epithelial cells can be found in Supplemental Table S1B where *Krt18* in CO epithelium is 1.77-fold increased over all other cell types, and *Krt18* in DES epithelium is 1.91-fold increased, indicating that there are similar levels of *Krt18* in both groups. This finding suggests that while many epithelial gene markers are expressed at a lower level in DES uteri, not all of them are. Comparisons of genes of interest can be done using Table S1B.

Fig. 1A and 2: CON should be replaced by CO (as used in text).

Response: We have replaced all instances of CON with CO.

Fig. 2A & C: In our opinion the tissue section used for the CO and DES condition are not of equal quality and are therefore not really comparable. For example, the tissue section used for the CO condition shows a nice, continuous muscle layer whereas the DES tissue section shows a discontinuous muscle layer. Should be explained.

Response: The quality of a section for spatial transcriptomics comes from the number of spots, number of genes per spot, number of reads, and median genes per spot. For all these parameters, CO and DES sections are quite similar (Lines 165-167). The cluster analysis allows us to separate cells into their respective cell types regardless of where they come from on the slide so any variation between the section morphology has less influence on the data.

It is not surprising that the DES tissue section has very little muscle evident. DES uteri are quite hypoplastic and have very thin muscle layers that can separate easily from the rest of the uterus during cryosectioning (see H&E images in Supplemental Figure S7A).

We have also now included spatial transcriptomics analysis on a second tissue section from both CO and DES, which gave us similar results. We have added these data to the Results (Lines 159-225) and to Supplemental Fig. S2A-C.

Fig. 3B, F, Fig. 4B: Include scalebars of feature plots to enable reliable comparison of expression levels between CO and DES.

Response: We have now included scale bars for the feature plots as suggested.

Fig. 3E: FOXA2 signal in not really clear in the LE (is very weak). Authors should provide better stainings/pictures.

Response: We have added two additional high magnification pictures in Fig. S3C to show FOXA2+ LE cells. We have also added the reference, Hancock, 2023 which shows sporadic FOXA2+ LE cells. The weak staining we observed in the LE corresponds to the lower mRNA levels found in CO cluster 11 compared to the mature GE (CO cluster 13) – see *Foxa2* violin plot in Fig. 3C.
