# Supplementary material for: Developmental estrogen exposure in mice disrupts uterine epithelial cell differentiation and causes adenocarcinoma via Wnt/β-catenin and PI3K/AKT signaling
Source: PLoS Biol. 2023 Oct 19;21(10):e3002334. doi: 10.1371/journal.pbio.3002334 (PMC10586657; doi:10.1371/journal.pbio.3002334)
Supplement: Attachment — Submitted filename: Response to Editor and Academic Editor.docx [file pbio.3002334.s023.docx]

Response to Editor and Academic Editor

Regarding the title, I think your proposed title change is fine, although we might suggest a

further modification, if you agree:

""Developmental estrogen exposure in mice disrupts uterine epithelial cell differentiation and causes adenocarcinoma via Wnt/β-catenin and PI3K/AKT signaling"

Response: The title was changed as requested above.

In light of our new Academic Editor's input, we think that only very minor changes will be required before publication. Specifically, please just address my editorial requests, regarding the ethics statement, and the western blot data (I have checked the GEO data and it seems to meet our requirements).

Response: The Ethics statement was changed as requested (lines 776-780)[Here and below, all line numbers refer to line numbers in the “track changes” version of the manuscript.]

The original western blots related to Figure 7H will be uploaded with the revised manuscript.

Note that I have now deleted the GEO token from the manuscript, as it should not be in the final manuscript. For your information, the token is: gtcjmyqorzynzkb.

As an additional editorial note, which I forgot to include in my previous leger, if any code was generated needed to support the conclusions of your manuscript, we would require that you make it available without restrictions upon publication. Please ensure that the code is sufficiently well documented and reusable, and that your Data Statement in the Editorial Manager submission system accurately describes where your code can be found.

Response: No original code was generated. Software used to analyze our data are listed in the Methods and are all publicly available.

While addressing these editorial requests are our only requirements for publication, we (the PLOS Biology editorial team) would still encourage you to give the manuscript a final readthrough, with our broad readership in mind. We suggest that you may even consider running your manuscript by a colleague who does not work in this field. While the manuscript is clearly accessible and of interest to experts working on this topic, streamlining of results and discussion is a request aimed at making the piece more accessible to a general readership, and we think this feedback from our previous Academic Editor should be considered, to the extent that is feasible, without losing essential information.

Response: We read through the full manuscript several times and got input from a non-expert. Doing so enabled us to shorten and streamline the text for clarity, and to provide better introductory sentences for a couple of paragraphs that pointed better to the focus of that paragraph. We also shortened the Discussion by a paragraph and simplified the graphical abstract. While I won’t detail every change here, as they are all available in the “track changes” version of the manuscript, the highlights are as follows:

1. Removed Pim3 violin plot from Figure S3B, as it did not fit exactly with the other 3 violin plots in that category. (see line 292)

2. Removed the feature plots of Top2a and Mki67 that included both the luminal and glandular epithelial cells from Figure 5B, leaving only the luminal cell feature plots. (see lines 385-386, 395-397, and 412-413)

3. Consolidated information regarding identification of DES cluster 5 as the DES cancer cluster into one paragraph (lines 536-544; moved up from lines 596-604). Switched order of panels 7C and 7D to accommodate this change. (lines 552-563 in Figure 7 legend)

4. Removed reference to loss of PTEN activity as cause of increased PI3K activity, as we did not test this idea directly (lines 606-607 and 613-614).

5. Deleted Conclusions paragraph as it was nonessential and somewhat redundant with Abstract (lines 760-769).

Figure changes

Figure 1 – Changed CON to CO

Figure 5 – Removed the feature plots of Top2a and Mki67 that included both the luminal and glandular epithelial cells from Figure 5B.

Figure 7 - Switched order of panels 7C and 7D

Figure 8 – Added basal cell image to legend

Figure S3B – Removed Pim3 violin plots and changed panel E label to CO12 markers from EpSC markers

Figure S4A – Added arrows to gene lists from Venn diagram numbers
